# *Foveapeltis* gen. nov., an unusual cleroid genus with large hypomeral cavities from mid‐Cretaceous amber (Coleoptera: Cleroidea)

**DOI:** 10.1002/ece3.11589

**Published:** 2024-07-08

**Authors:** Yan‐Da Li, Jiří Kolibáč, Zhen‐Hua Liu, Adam Ślipiński, Shûhei Yamamoto, Ya‐Li Yu, Wei‐Ting Zhang, Chen‐Yang Cai

**Affiliations:** ^1^ State Key Laboratory of Palaeobiology and Stratigraphy, Nanjing Institute of Geology and Palaeontology Chinese Academy of Sciences Nanjing China; ^2^ Bristol Palaeobiology Group, School of Earth Sciences University of Bristol Bristol UK; ^3^ Department of Entomology Moravian Museum Brno Czech Republic; ^4^ Guangdong Key Laboratory of Animal Conservation and Resource Utilization, Guangdong Public Laboratory of Wild Animal Conservation and Utilization, Institute of Zoology Guangdong Academy of Sciences Guangzhou China; ^5^ Australian National Insect Collection CSIRO Canberra Australian Capital Territory Australia; ^6^ Hokkaido University Museum Hokkaido University Sapporo Japan; ^7^ Institute of Paleontology Hebei GEO University Shijiazhuang China

**Keywords:** Cleroidea, Cretaceous, fossil, Kachin amber, parsimony, phylogenetic analysis

## Abstract

Beetles have a remote evolutionary history dating back to the Carboniferous, with Mesozoic fossils playing a pivotal role in elucidating the early evolution of extant families. Despite their exceptional preservation in amber, deciphering the systematic positions of Mesozoic trogossitid‐like beetles remains challenging. Here, we describe and illustrate a new trogossitid‐like lineage from mid‐Cretaceous Kachin amber, *Foveapeltis rutai* Li, Kolibáč, Liu & Cai, gen. et sp. nov. *Foveapeltis* stands out within the Cleroidea due to the presence of a significant large cavity on each hypomeron. While the exact phylogenetic placement of *Foveapeltis* remains uncertain, we offer a discussion on its potential affinity based on our constrained phylogenetic analyses.

## Graphical abstract

Deciphering the systematic positions of Mesozoic trogossitid‐like beetles is challenging. Here, we describe and illustrate a new trogossitid‐like lineage from mid‐Cretaceous Kachin amber, *Foveapeltis rutai* gen. et sp. nov. *Foveapeltis* stands out within the Cleroidea due to the presence of a significant large cavity on each hypomeron. While the exact phylogenetic placement of *Foveapeltis* remains uncertain, we offer a discussion on its potential affinity based on our constrained phylogenetic analyses.

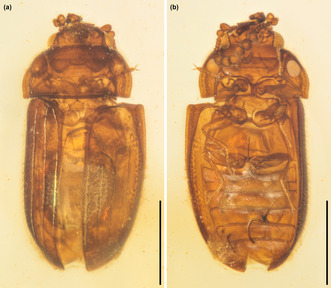

## 1 INTRODUCTION

Cleroidea is a moderately diverse superfamily of cucujiform beetles, with 18 families as recognized by Gimmel et al. (2019). Within Cleroidea, the partly soft‐bodied melyrid lineage has been well recognized as monophyletic, including Phycosecidae, Prionoceridae, Mauroniscidae, Rhadalidae and Melyridae. The clerid lineage includes Thanerocleridae and Cleridae, and probably also Chaetosomatidae (Gimmel et al., 2019; Kolibáč et al., 2021; Li et al., 2021). Except for a few basal lineages (Byturidae, Biphyllidae and Acanthocnemidae), the remaining cleroids have all been classified at one time in a broadly defined Trogossitidae (e.g., Kolibáč, 2013). The internal classification of this broadly defined Trogossitidae was often inconsistent among different morphology‐based studies (Kolibáč, 2013: table 1; Gimmel et al., 2019: appendix 1B). Molecular evidence suggested that the aforementioned Trogossitidae sensu Kolibáč (2013) would not be monophyletic (e.g., Gimmel et al., 2019; McKenna et al., 2019). The family has been formally split into Rentoniidae, Phloiophilidae, Protopeltidae, Peltidae, Lophocateridae, Trogossitidae sensu stricto and Thymalidae by Gimmel et al. (2019), although the circumscription of some of these families may still require further revision. Hereafter, Trogossitidae is used in its modern sense following Gimmel et al. (2019) unless otherwise specified, and the taxa of Trogossitidae sensu Kolibáč (2013) are referred to as trogossitid‐like groups.

Several Mesozoic genera preserved as adpression fossils have at least once been associated with the trogossitid‐like groups (Kolibáč, 2013; Schmied et al., 2009, 2011). However, their familial attribution is often difficult to evaluate due to the poor state of preservation, especially considering that the trogossitid‐like families redefined by Gimmel et al. (2019) often lack clear and easy‐to‐observe apomorphies (e.g., Kolibáč, 2013; Yu et al., 2014, 2015). Recently discovered amber fossils preserve greater details facilitating classification; nevertheless, the mixture of diagnostic characters of different families still makes the familial assignment thorny in some cases. *Cretamerus* Peris et al. from mid‐Cretaceous French amber was suggested to be possibly related to Decamerini (Thymalidae), although this hypothesis was not unequivocally demonstrated by their cladistic analysis (Peris et al., 2014). *Burmacateres* Kolibáč & Peris, *Gracilenticrus* Yu et al., *Parayixianteres* Yu et al. and *Zaiwa* Lyubarsky et al. from mid‐Cretaceous Kachin amber of northern Myanmar were assigned to Lophocateridae (Kolibáč & Peris, 2021; Lyubarsky et al., 2021; Yu, Leschen, et al., 2021; Yu, Li, et al., 2021). In the case of *Gracilenticrus* where a cladistic analysis was done, however, the assignment to Lophocateridae was not proved (Yu, Li, et al., 2021). *Zaiwa* also exhibits features unknown among the extant lophocaterids, making its placement suspicious. *Microtrogossita* Li & Cai from Kachin amber was assigned to Trogossitidae as demonstrated by a cladistic analysis, although it still possesses some unusual features such as the relatively widely separated pro‐ and mesocoxae (Li et al., 2021).

Here, we describe another unusual group of cleroid fossils from mid‐Cretaceous Kachin amber, which in body shape resembles some members of the trogossitid‐like families, but bears characters hitherto unknown in the whole Cleroidea.

## 2 MATERIALS AND METHODS

### 2.1 Materials

**FIGURE 1:**
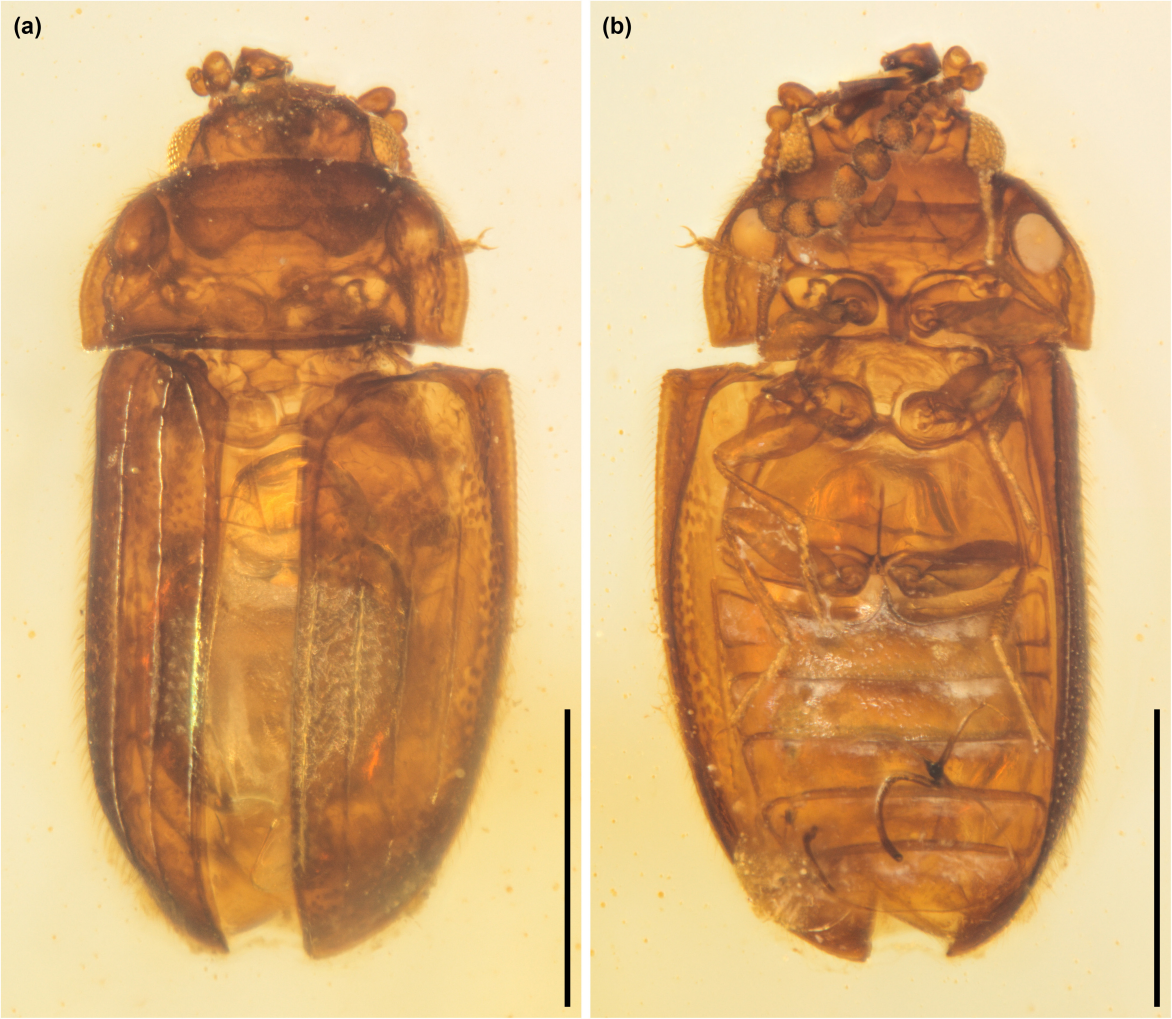
General habitus of *Foveapeltis rutai* Li, Kolibáč, Liu & Cai, gen. et sp. nov., holotype, NIGP203568, under incident light. (a) Dorsal view; (b) Ventral view. Scale bars: 500 μm.

**FIGURE 2:**
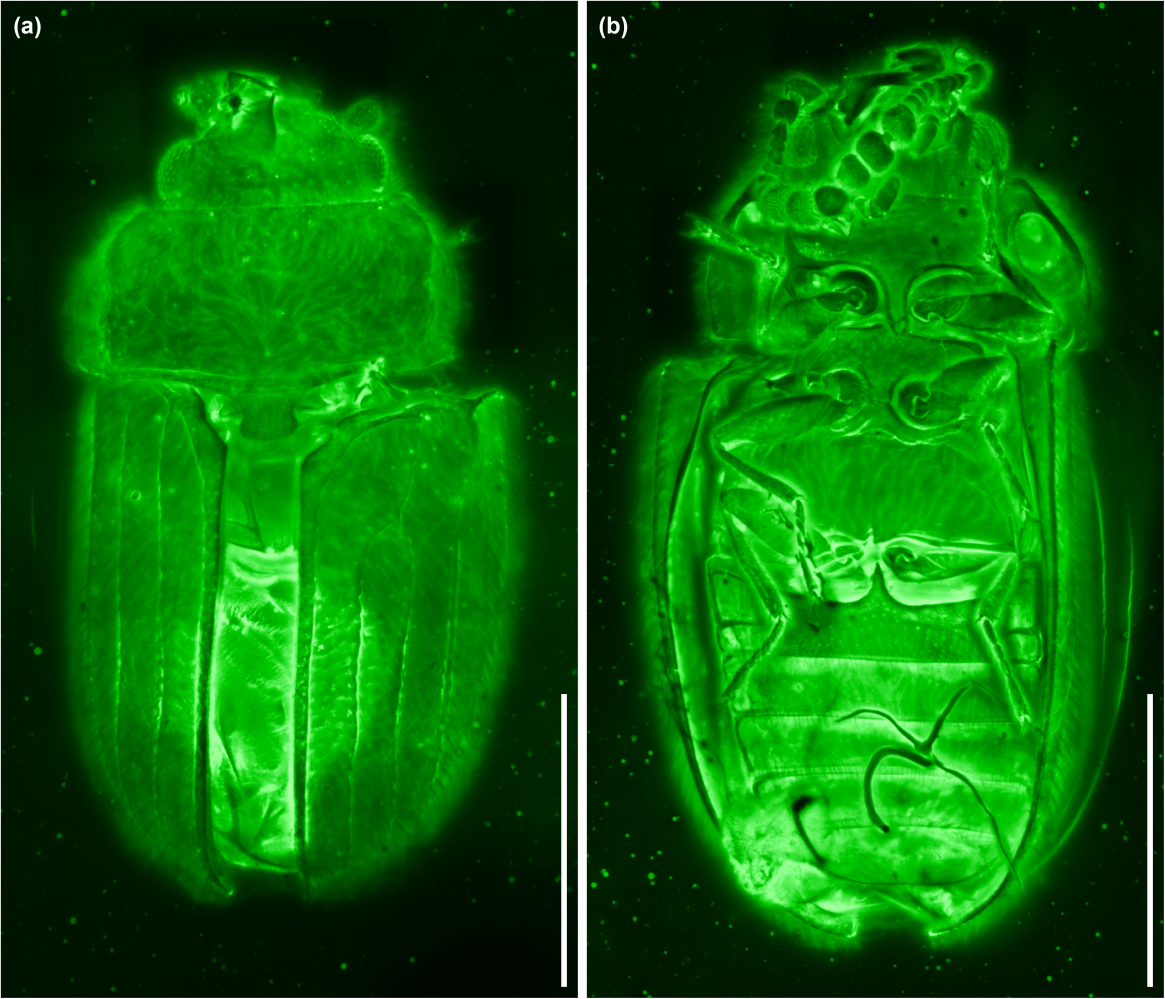
General habitus of *Foveapeltis rutai* Li, Kolibáč, Liu & Cai, gen. et sp. nov., holotype, NIGP203568, under confocal microscopy. (a) Dorsal view; (b) Ventral view. Scale bars: 500 μm.

**FIGURE 3:**
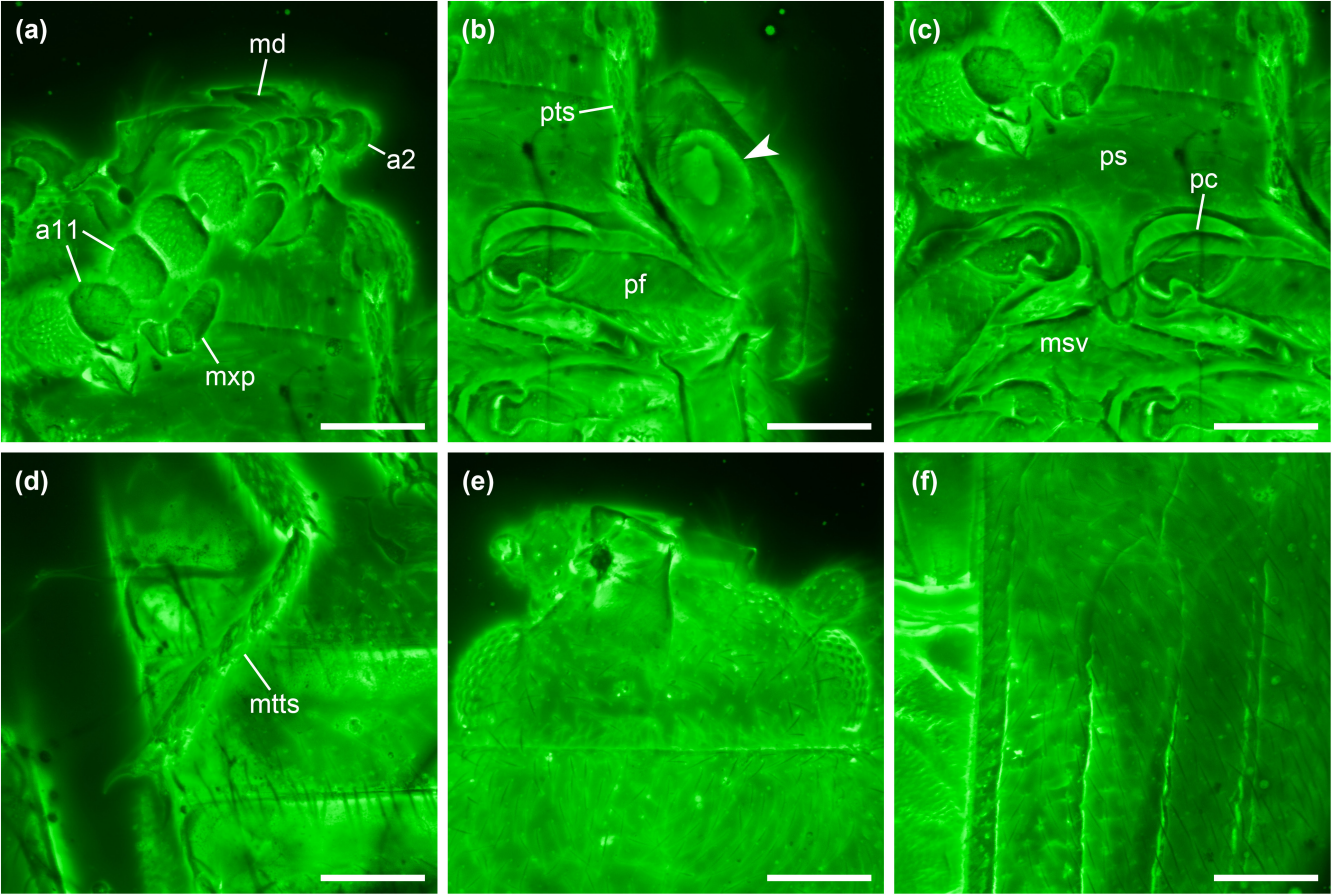
Details of *Foveapeltis rutai* Li, Kolibáč, Liu & Cai, gen. et sp. nov., holotype, NIGP203568, under confocal microscopy. (a) Head, ventral view; (b) Hypomeral cavity (arrowhead), ventral view; (c) Prothorax, ventral view; (d) Metatarsus, dorsal view; (e) Head, dorsal view; (f) Elytron, dorsal view. Abbreviations: a2–11, antennomeres 2–11; md, mandible; msv, mesoventrite; mtts, metatarsus; mxp, maxillary palp; pc, procoxa; pf, profemur; ps, prosternum; pts, protarsus. Scale bars: 100 μm.

**FIGURE 4:**
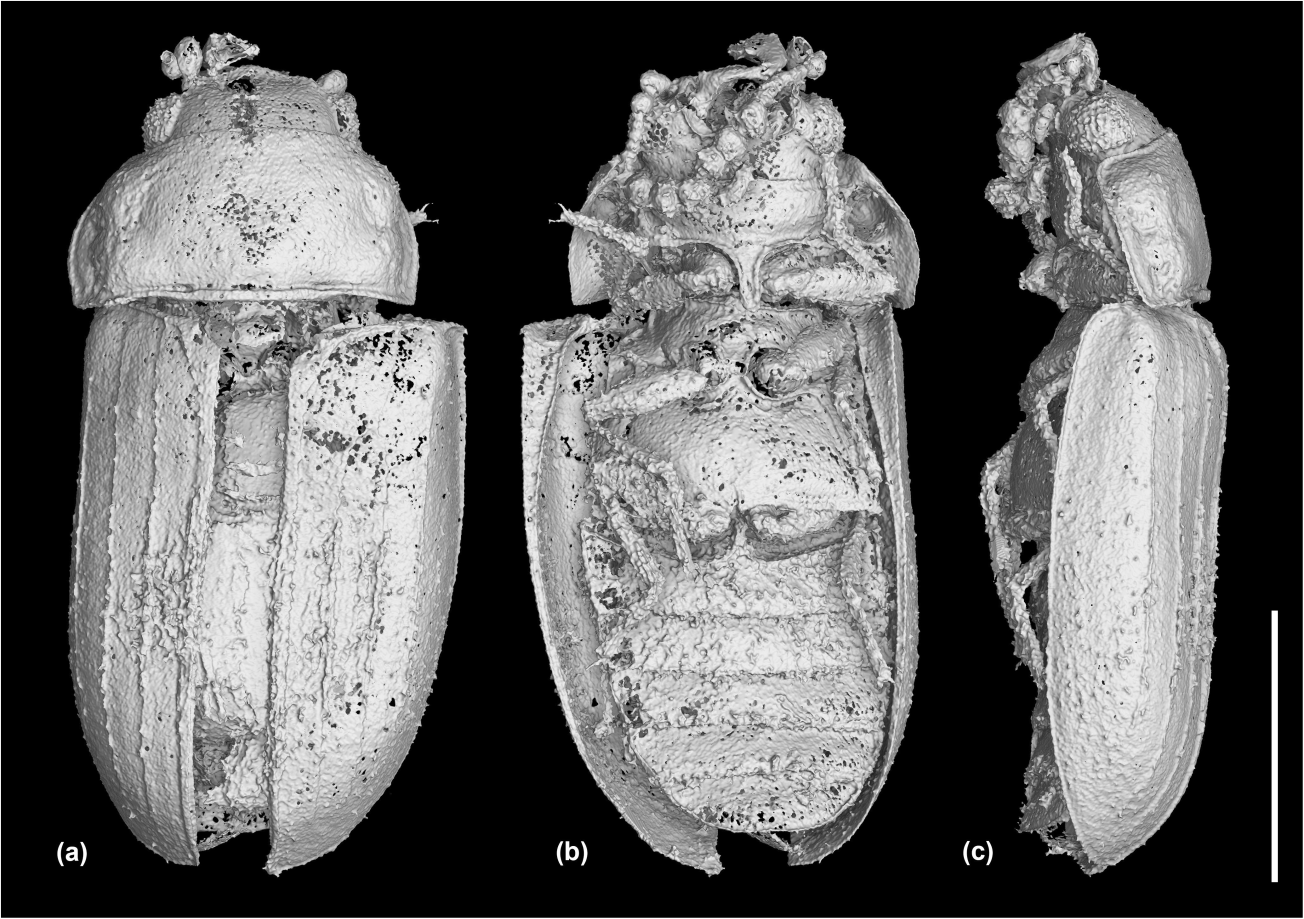
X‐ray microtomographic reconstruction of *Foveapeltis rutai* Li, Kolibáč, Liu & Cai, gen. et sp. nov., holotype, NIGP203568. (a) Dorsal view; (b) Ventral view; (c) Lateral view. Scale bar: 500 μm.

**FIGURE 5:**
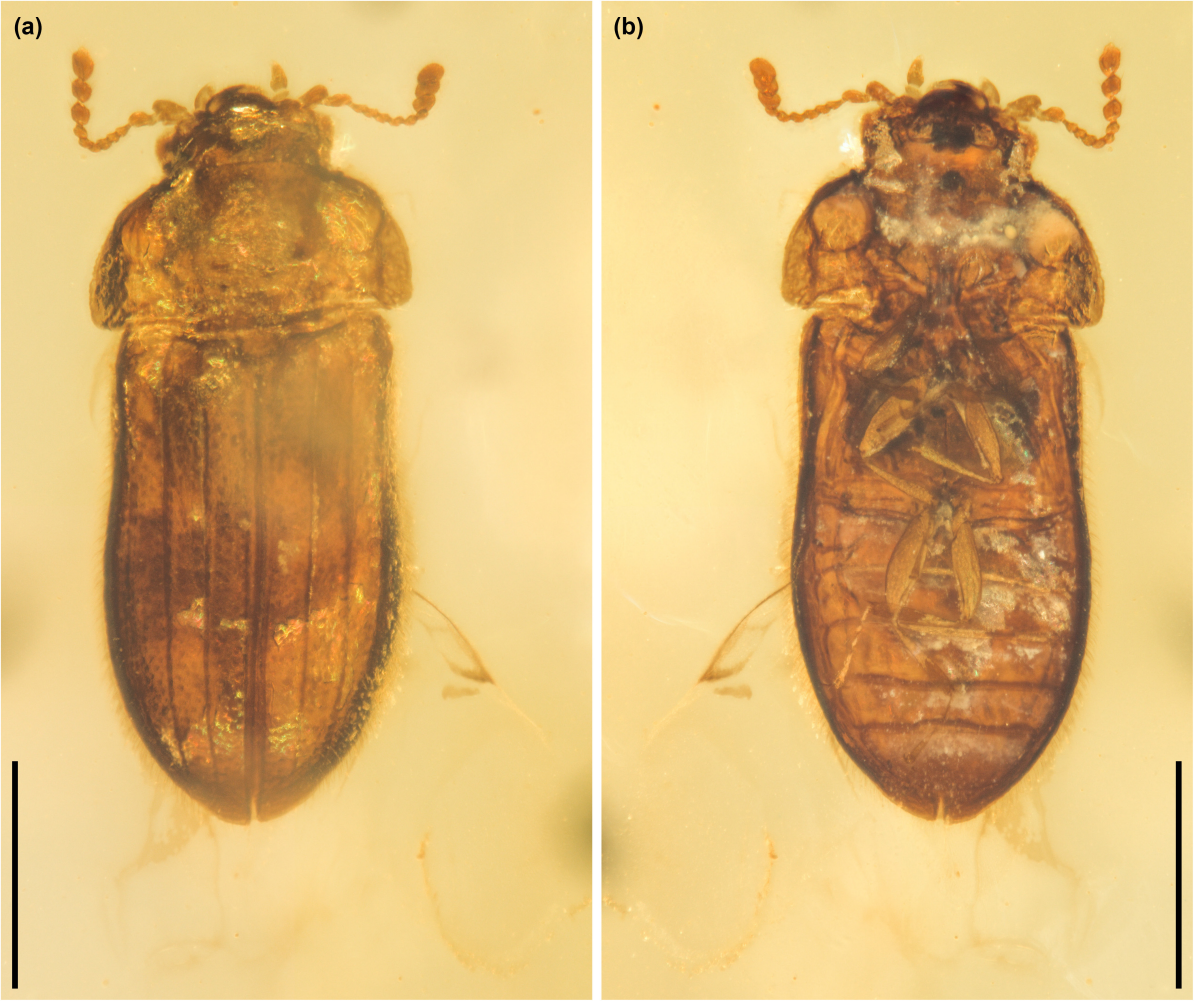
General habitus of *Foveapeltis rutai* Li, Kolibáč, Liu & Cai, gen. et sp. nov., paratype, IZGAS‐BA‐COL001, under incident light. (a) Dorsal view; (b) Ventral view. Scale bars: 500 μm.

**FIGURE 6:**
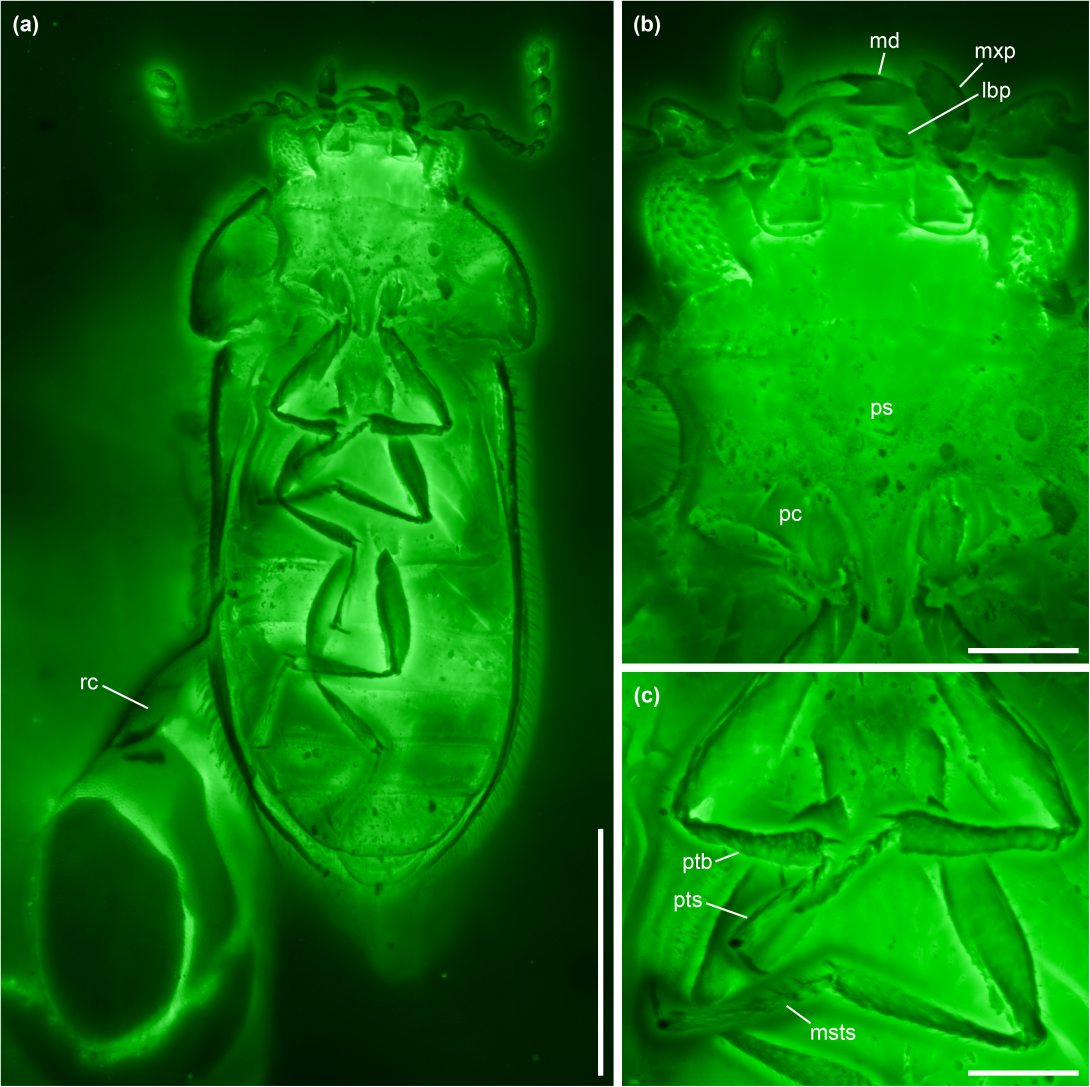
*Foveapeltis rutai* Li, Kolibáč, Liu & Cai, gen. et sp. nov., paratype, IZGAS‐BA‐COL001, under confocal microscopy. (a) Habitus, ventral view; (b) Head and prothorax, ventral view; (c) Fore and mid legs. Abbreviations: msts, mesotarsus; pc, procoxa; ps, prosternum; ptb, protibia; pts, protarsus; rc, radial cell. Scale bars: 500 μm in (a), 100 μm in (b, c).

**FIGURE 7:**
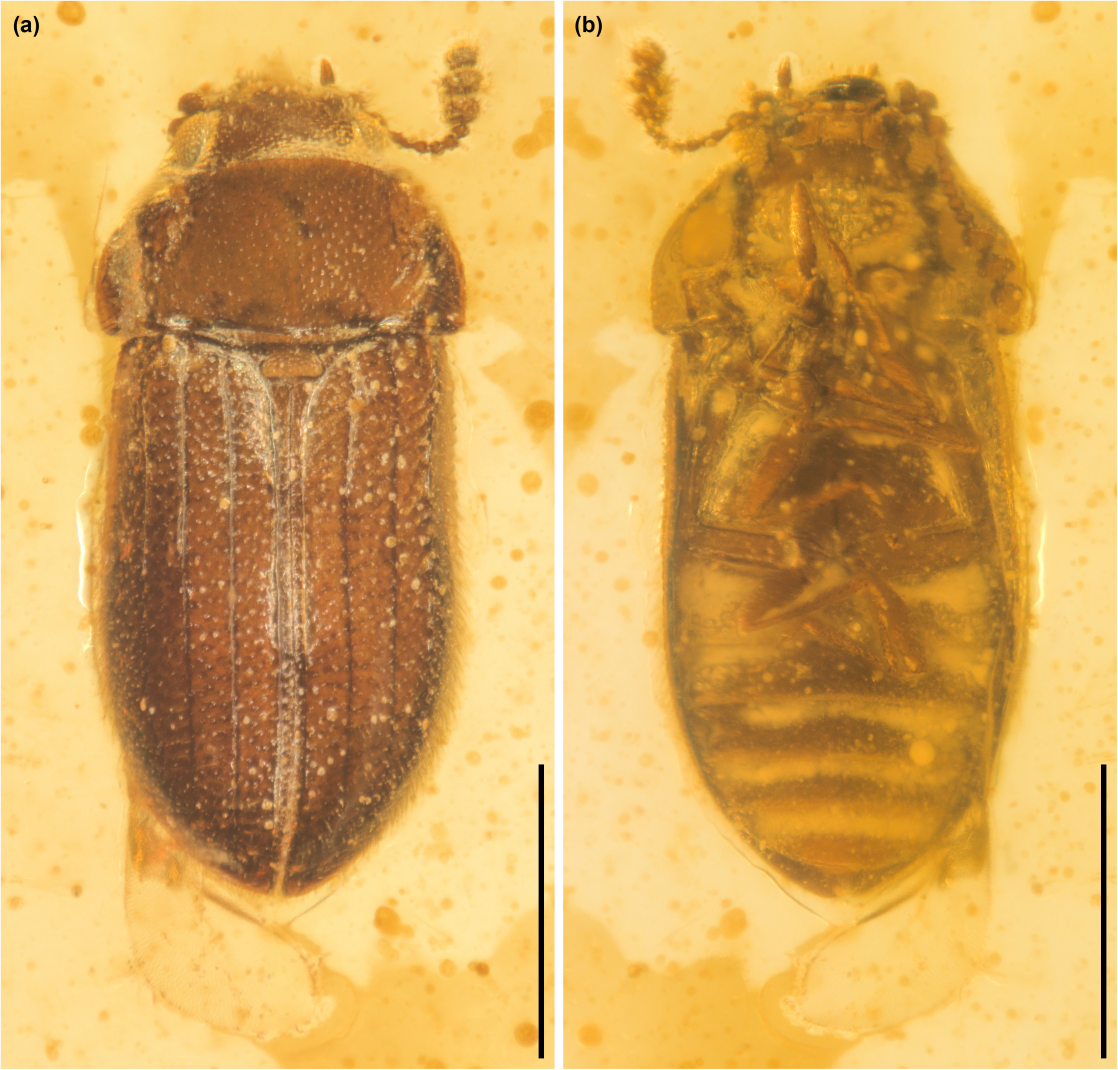
General habitus of *Foveapeltis rutai* Li, Kolibáč, Liu & Cai, gen. et sp. nov., paratype, IZGAS‐BA‐COL003, under incident light. (a) Dorsal view; (b) Ventral view. Scale bars: 500 μm.

**FIGURE 8:**
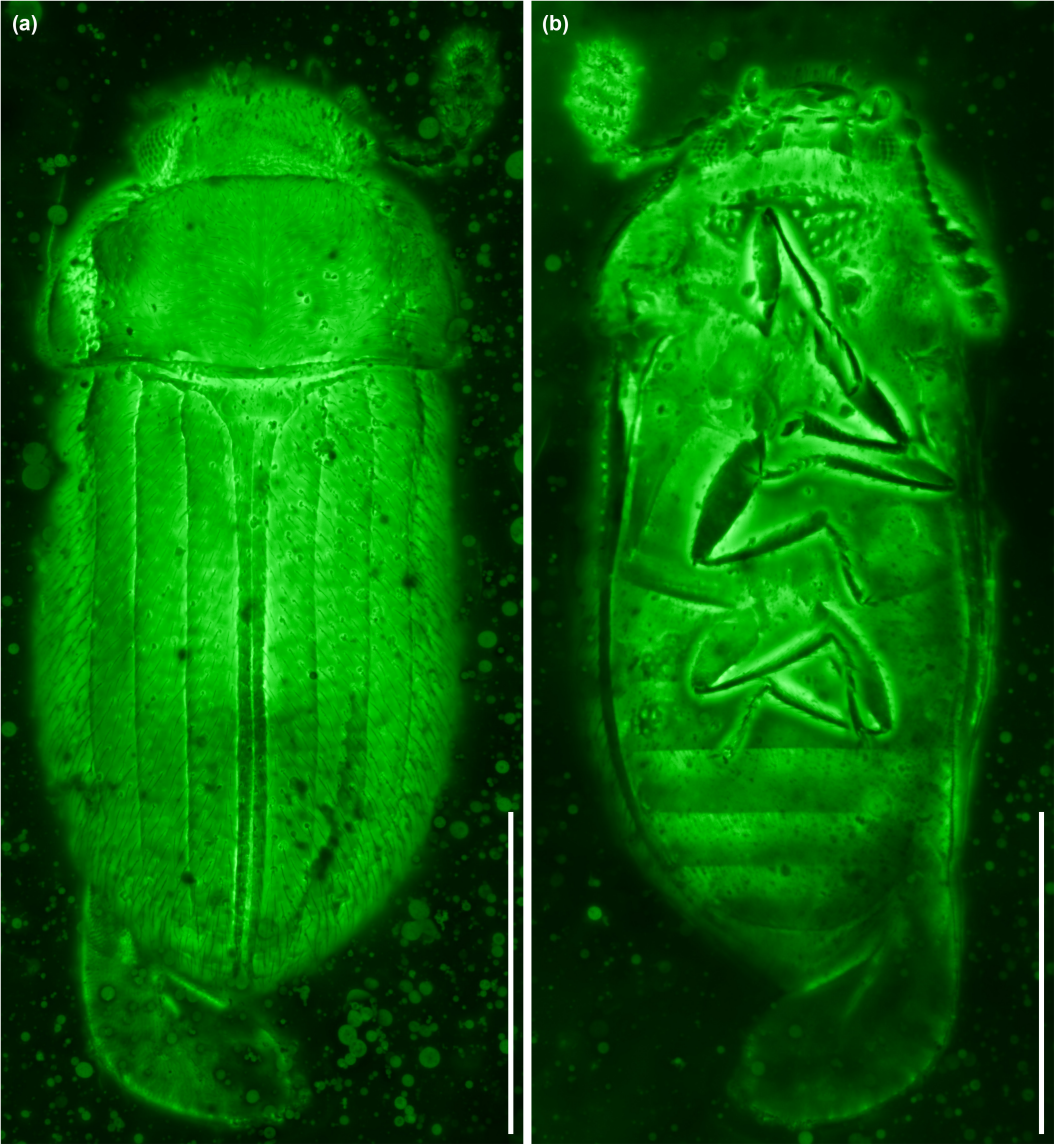
General habitus of *Foveapeltis rutai* Li, Kolibáč, Liu & Cai, gen. et sp. nov., paratype, IZGAS‐BA‐COL003, under confocal microscopy. (a) Dorsal view; (b) Ventral view. Scale bars: 500 μm.

**FIGURE 9:**
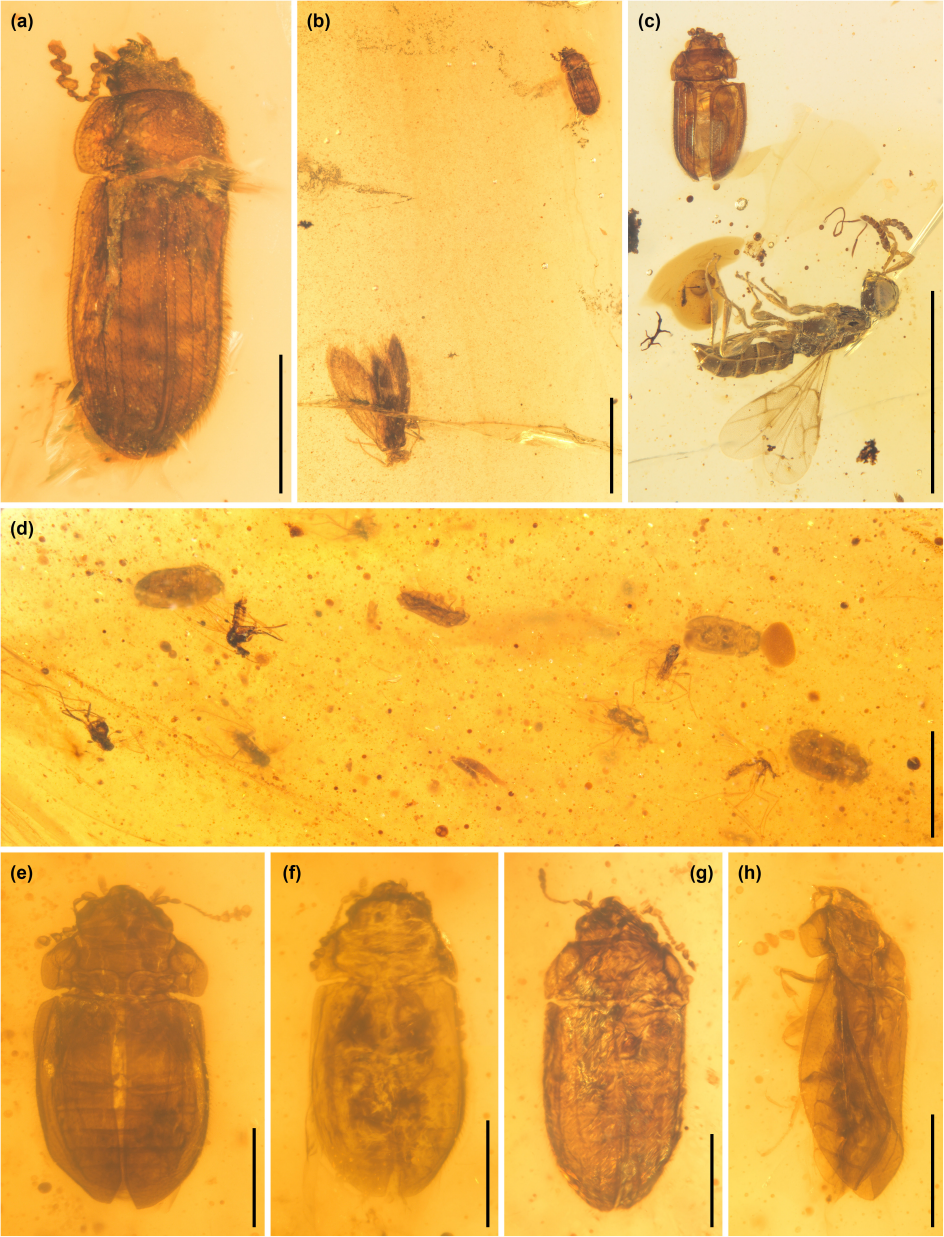
Syninclusions of *Foveapeltis*. (a) *Foveapeltis rutai* Li, Kolibáč, Liu & Cai, gen. et sp. nov., paratype, IZGAS‐BA‐COL002; (b) *Foveapeltis rutai*, IZGAS‐BA‐COL002, preserved along with a moth (Micropterigidae); (c) *Foveapeltis rutai*, NIGP203568, preserved along with a parasitoid wasp (Platygastroidea nr. Proterosceliopsidae); (d) IZGAS‐BA‐COL004, four individuals of *Foveapeltis rutai* preserved along with sand flies (Psychodidae: Sycoracinae); (e) IZGAS‐BA‐COL004‐1; (f) IZGAS‐BA‐COL004‐2; (g) IZGAS‐BA‐COL004‐3; (h) IZGAS‐BA‐COL004‐4. Scale bars: 500 μm in (a, e–h), 2 mm in (b–d).

The Kachin amber (Burmese amber) specimens studied herein (Figures 1, 2, 3, 4, 5, 6, 7, 8, 9) originated from amber mines near Noije Bum (26°20′ N, 96°36′ E), Hukawng Valley, Kachin State, northern Myanmar. The specimen NIGP203568 is deposited in the Nanjing Institute of Geology and Palaeontology (NIGP), Chinese Academy of Sciences, Nanjing, China. The remaining specimens (four amber pieces) are deposited in the Institute of Zoology, Guangdong Academy of Sciences (IZGAS), Guangzhou, China. When necessary, the amber pieces were trimmed with a small table saw, ground with emery papers of different grit sizes and finally polished with polishing powder.

### 2.2 Fossil imaging

Brightfield images were taken with a Zeiss Discovery V20 stereo microscope. Confocal images were obtained with a Zeiss LSM710 confocal laser scanning microscope, using the 488 nm Argon laser excitation line (Fu et al., 2021). Images were stacked with Helicon Focus 7.0.2, Zerene Stacker 1.04 and Adobe Photoshop CC, and were further processed in Adobe Photoshop CC to adjust brightness and contrast. Microtomographic data for the specimen NIGP203568 were obtained with a Zeiss Xradia 520 Versa 3D X‐ray microscope at the micro‐CT laboratory of NIGP and analyzed in VGStudio MAX 3.0. Scanning parameters were as follows: isotropic voxel size, 1.7815 μm; power, 3 W; acceleration voltage, 40 kV; exposure time, 4 s; projections, 3001.

### 2.3 Description and measurement

The morphological terminology generally follows Lawrence and Ślipiński (2013) and Kolibáč (2005, 2013). It should be noted that the position of the mandibular apical teeth in the horizontal or vertical axis always refers to the configuration in dorsal/ventral view in Kolibáč (2005, 2006), while in some other coleopteran publications (e.g., Escalona et al., 2020; Hörnschemeyer, 2009) it refers to the configuration in apical view. *Microtrogossita* actually has mandibular teeth horizontally situated in dorsal/ventral view, and was incorrectly coded in the analysis by Li et al. (2021).

The length and width of body parts are affected by the viewing angle and the conformation of the beetle body. Therefore, here only the body length (BL) and body width (BW) are provided as rough indicators of the overall body size. The measurements of body length were taken as the apparent distance from mandibular apex to elytral apex in dorsal view. The measurements of body width were taken as the pronotal width, even though in the specimen NIGP203568 the width across the detached elytra appears to be superficially larger.

### 2.4 Phylogenetic analyses

As shown by previous studies (e.g., Gimmel et al., 2019; Kolibáč, 2006; Li et al., 2021), the phylogenetic relationships among the trogossitid‐like groups cannot be properly resolved based on morphological information alone (i.e., unconstrained morphology‐based analyses). Thus, to evaluate the systematic placement of the new fossil genus, we conducted constrained morphology‐based phylogenetic analyses under maximum parsimony. The use of molecular‐based constraints would allow a more realistic estimation of the states at ancestral nodes, and therefore contribute to a more authentic placement of the fossil (Fikáček et al., 2020). The data matrix for extant genera was taken from Li et al. (2021), which was derived from Kolibáč (2006, 2008). The full matrix includes 61 adult and 32 larval characters, among which we successfully coded 31 adult characters for the new fossil (File S1). The constraining backbone tree was created based on the Bayesian molecular tree by Li et al. (2021), who re‐analyzed Gimmel et al.'s (2019) data with the site‐heterogeneous model CAT‐GTR + G4. Site‐heterogeneous models could generally improve the accuracy of phylogenetic analyses (e.g., Li, Engel, et al., 2023), and specifically, compared with Gimmel et al. (2019), the result by Li et al. (2021) is more accordant to the phylogenomic studies by McKenna et al. (2019) and Cai et al. (2022). It should be nevertheless noted that the result by Li et al. (2021) is still not fully compatible with the phylogenomic studies. However, the results of these phylogenomic studies were not directly used here due to the much sparser taxon sampling.

The parsimony analyses were performed under both equal and implied weights, using R 4.1.0 (R Core Team, 2021) and the R package TreeSearch 1.3.1 (Smith, 2023). The concavity constant in the weighted analyses was set to 12, following the suggestion by Goloboff et al. (2018) and Smith (2019).

In the first analysis (File S2), all taxa in the morphological matrix were included. For taxa with both morphological and molecular data, their interrelationships were fixed as the backbone tree. The fossil genus and other extant taxa without molecular data were allowed to move freely across the backbone tree (e.g., Li, Liu, et al., 2023; Li, Newton, et al., 2022; Li, Ślipiński, et al., 2023; Li, Yamamoto, et al., 2023; Li, Zhang, et al., 2022). The resulting tree was visualized with the online tool iTOL 6.6 (Letunic & Bork, 2024) and graphically edited with Adobe Illustrator CC 2017.

In the second set of analyses (File S3), only the taxa present in the backbone tree and the fossil genus were included. Only the fossil genus was allowed to move freely across the backbone tree. In order to perceive the uncertainty of the fossil placement, the parsimony scores of the trees with alternative placements of the fossil were mapped to the corresponding branches of the backbone tree (Li et al., 2024). The results were visualized with the R package ggtree 6.5.2 (Yu, 2020; Yu et al., 2017) and graphically edited with Adobe Illustrator CC 2017.

## 3 SYSTEMATIC PALEONTOLOGY

Order Coleoptera Linnaeus, 1758

Superfamily Cleroidea Latreille, 1802

Family *incertae sedis*

### 3.1 Genus *Foveapeltis* Li, Kolibáč, Liu & Cai, gen. nov.

**Type species.** *Foveapeltis rutai* sp. nov.

**Etymology.** The generic name is formed based on the Latin “*fovea*,” pit, referring to the large hypomeral cavities, and *Peltis* Müller, a well‐known cleroid genus, referring to the overall similar habitus. The name is feminine in gender.

**Diagnosis.** Frontoclypeal suture absent (Figure 3e). Antennae 11‐segmented; club 3‐segmented, symmetrical (Figure 3a). Mandibles with two apical teeth vertically situated in apical view (Figures 3a and 6b). Pronotal hypomera each with a large cavity (Figure 3b). Procoxal cavities strongly transverse, externally open; hypomeral postcoxal process longer than half transverse procoxal diameter (Figures 3c and 6b). Procoxae not projecting (Figures 3c and 6b). Elytra with three distinct longitudinal carinae (Figures 5a and 8a); punctures moderate in size and irregularly arranged (Figure 3f); epipleura narrow and incomplete (Figures 2b, 4b, and 6a). Hind wings with oblong radial cell (Figures 5 and 6a). All tibiae without conspicuous spines along outer margin, with two unhooked spurs at apex (Figures 3b and 6c).

### 3.2 *Foveapeltis rutai* Li, Kolibáč, Liu & Cai, sp. nov. (Figures 1, 2, 3, 4, 5, 6, 7, 8, 9)

**Material.** Holotype, NIGP203568 (Figures 1, 2, 3, 4). Paratypes, IZGAS‐BA‐COL001 (Figures 5 and 6), IZGAS‐BA‐COL002 (Figure 9a), IZGAS‐BA‐COL003 (Figures 7 and 8).

**Etymology.** The species is named after the coleopterist Dr. Rafał Ruta (University of Wrocław, Poland), an expert especially in the systematics of Scirtidae.

**Locality and horizon.** Amber mine located near Noije Bum Village, Tanai Township, Myitkyina District, Kachin State, Myanmar; unnamed horizon, mid‐Cretaceous, Upper Albian to Lower Cenomanian.

**Diagnosis.** As for the genus (vide supra).

**Description.** Body minute, oval, moderately convex; surface with fine semierect hairs.

Head prognathous, including eyes narrower than anterior margin of pronotum. Frons simple, without any grooves or processes. Frontoclypeal suture absent. Compound eyes moderately large, not emarginate, laterally situated. Antennal grooves absent. Antennae with 11 antennomeres; antennomeres 9–11 enlarged, symmetrical, forming distinct club. Mandibles with two apical teeth vertically situated in apical view (horizontally situated in dorsal/ventral view). Maxillary palps 4‐segmented; apical palpomere subconical. Ventral side of head without any tufts of setae. Gular sutures inconspicuous.

Pronotal disc transverse, slightly narrowing anteriorly, slightly wider at base than combined elytral bases; anterior edge nearly straight in dorsal view; anterior and posterior corners nearly right‐angled in lateral view; lateral edges smooth. Hypomeron each with a large cavity. Prosternum in front of coxae transverse; prosternal process complete, not dilated at apex; apex rounded. Procoxal cavities strongly transverse, separated by about half longitudinal diameter of coxae, externally open; hypomeral postcoxal process longer than half transverse procoxal diameter. Procoxae not projecting.

Scutellar shield small, transverse, anteriorly not abruptly elevated. Elytra completely covering abdomen, subparallel in basal half, tapering apically; surface with three distinct longitudinal carinae; punctures moderate in size and irregularly arranged; epipleura very narrow beyond humeral region, apically incomplete. Mesocoxal cavities weakly transverse, separated by about half longitudinal diameter of coxae, laterally open (bordered partly by mesepimeron). Metaventrite broad; discrimen present posteriorly; metakatepisternal suture absent; postcoxal lines absent. Exposed portion of metanepisternum elongate. Metacoxae very narrowly separated, laterally reaching elytral epipleura; coxal plates absent.

Hind wings with oblong radial cell.

All trochanters small, triangular. All tibiae without conspicuous spines along outer margin, with two unhooked spurs at apex. Tarsal formula 4‐4‐4 (tarsomere 1 relatively long, apparently fused with the formerly second tarsomere); tarsomeres simple, without appendages; tarsomere 4 at least as long as tarsomeres 1–3 in all pairs of legs. Pretarsal claws thickened at base (seemingly with weak denticle); empodium bisetose.

Abdomen with five ventrites; intercoxal process acute; ventrite 5 apically broadly rounded.

**Measurements.** NIGP203568: BL 1.52 mm, BW 0.65 mm. IZGAS‐BA‐COL001: BL 1.63 mm, BW 0.71 mm. IZGAS‐BA‐COL002: BL 1.52 mm, BW 0.63 mm. IZGAS‐BA‐COL003: BL 1.40 mm, BW 0.63 mm.

**Remarks.** In the right elytron of specimen NIGP203568, the two inner carinae merge together near the elytral base. We regard this as an individual variation, as its left elytron is the same as other specimens, with all carinae isolated. The specimen IZGAS‐BA‐COL003 appears to have a more distinctly punctured prosternum compared to NIGP203568 and IZGAS‐BA‐COL001. However, in extant beetles, the punctation could vary within one species. Thus, here we decide to not establish a separate species based on IZGAS‐BA‐COL003, especially considering that no other good differential characters have been found for it.

## 4 RESULTS

**FIGURE 10:**
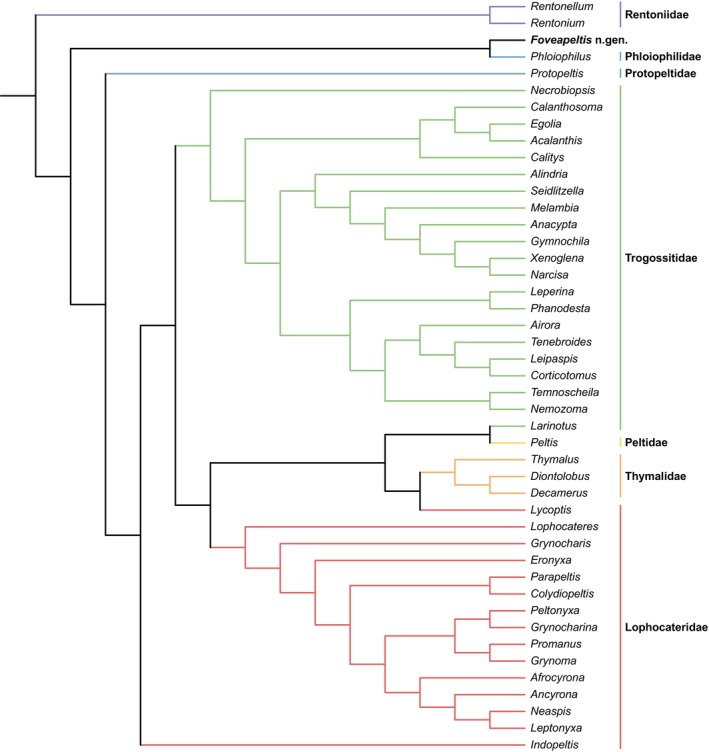
The most parsimonious placement of *Foveapeltis*, analyzed based on the full matrix. Tree resulting from the constrained parsimony analysis under implied weights.

**FIGURE 11:**
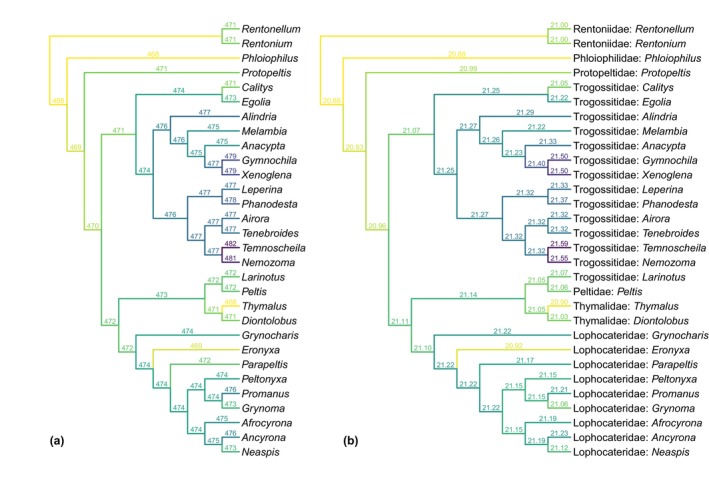
Constrained parsimony analyses showing alternative placements of *Foveapeltis*. The score above each branch represents the parsimony score of the topology in which *Foveapeltis* is inserted to that branch. (a) Analysis under equal weights. (b) Analysis under implied weights.

In the best tree under implied weights (Figure 10), *Foveapeltis* is resolved as the sister group of *Phloiophilus* (Phloiophilidae). As the best tree is not substantially better than the alternatives, it would be informative to also explore the suboptimal options. With the parsimony score of other possibilities labeled on the backbone tree, it appears that *Foveapeltis* may also be related to *Thymalus* (Thymalidae) or *Eronyxa* (Lophocateridae), or located in the basalmost part of the sampled taxa (Figure 11).

## 5 DISCUSSION

Within Cleroidea, *Foveapeltis* gen. nov. could be easily excluded from Byturidae and Biphyllidae by the metacoxae laterally meeting elytral epipleura and simple tarsi (Goodrich & Springer, 1992; Springer & Goodrich, 1986); from Acanthocnemidae by the wider and longer prosternal process and non‐projecting procoxae (Kolibáč, 2018; Matsumoto & Geiser, 2021); from the clerid lineage by the apically bidentate mandibles and strongly transverse procoxae (Crowson, 1970; Kolibáč, 2004; Kolibáč & Huang, 2016); and finally from the melyrid lineage by the non‐projecting procoxae (except for Phycosecidae) and distinctly 3‐segmented antennal club (Crowson, 1970; Gimmel et al., 2019; Lawrence et al., 2014; Ślipiński, 1992).

*Foveapeltis* differs from all the abovementioned lineages in terminal tarsomere approximately as long as other tarsomeres together and elytra with distinct longitudinal carinae. The long terminal tarsomere is shared with the remaining cleroid groups including Rentoniidae, Phloiophilidae, Protopeltidae, Trogossitidae, Thymalidae, Peltidae and Lophocateridae, which were all historically classified in a broadly circumscribed Trogossitidae (Kolibáč, 2013). *Foveapeltis* shares very small body size (1–2 mm) with Rentoniidae, but the latter group always has a conglobate body with smooth dorsal surface (Gimmel & Leschen, 2014; Lawrence & Ślipiński, 2013). The elytral carinae are present in some members of Trogossitidae and Lophocateridae. However, *Foveapeltis* could be ruled out from Trogossitidae based on its externally open procoxal cavites; as in Trogossitidae (including Larinotinae) the procoxal cavites are broadly closed externally (Kolibáč, 2013). *Foveapeltis* has symmetrical antennal clubs and no frontoclypeal suture, while Lophocateridae (except for *Colydiopeltis* Ślipiński and *Parapeltis* Ślipiński) is generally characterized by the presence of frontoclypeal suture (reduced in *Antillipeltis* Lawrence et al. and some *Ancyrona*‐related genera; Kolibáč, 2007, 2014; Lawrence et al., 2014), weakly asymmetrical antennal club and tibiae with spines along sides. The somewhat unusual *Colydiopeltis* and *Parapeltis* have 8‐segmented antennae, and *Parapeltis* additionally has externally closed procoxal cavites (Ślipiński, 1992). The strange *Antillipeltis* differs in structure of its lobate tarsi (Lawrence et al., 2014). Therefore, *Foveapeltis* is unlikely to belong to Lophocateridae as well.

The remaining cleroid groups are more or less similar to *Foveapeltis* in habitus. The monogeneric Peltidae shares with *Foveapeltis* the longitudinal elytral carinae, but differs from the latter in the larger body size, the presence of frontoclypeal suture, complete elytral epipleura, and protibiae with a large curved spur at apex and spines along outer edge (Kolibáč, 2013). Thymalidae as defined by Gimmel et al. (2019) includes Decamerinae and Thymalinae. The adults of Decamerinae are morphologically similar to Lophocateridae, also characterized by the presence of frontoclypeal suture, weakly asymmetrical antennal club and tibiae with spines (Kolibáč, 2013). They moreover have toothed tarsal claws, therefore obviously differ from *Foveapeltis*. Thymalinae, and also Protopeltidae and Phloiophilidae, share with *Foveapeltis* the symmetrical antennal club, externally open procoxal cavites and the absence of frontoclypeal suture. Protopeltidae can be differentiated from *Foveapeltis* in the lateral sides of protibiae with spines and elytral epipleura complete (Crowson, 1964); Phloiophilidae can be differentiated from *Foveapeltis* in the procoxae slightly projecting (Kolibáč, 2008); Thymalinae can be differentiated from *Foveapeltis* in the body strongly convex, head mostly covered by pronotum and elytral epipleura wide and complete (Asakawa et al., 2020; Asakawa & Yoshitomi, 2019). Besides, the elytra of Thymalidae, Protopeltidae and Phloiophilidae are never carinate. Phloiophilidae is possibly the morphologically most ancestral family among the mentioned extant taxa as indicated by the absence of hooked spur on the protibia and also characteristics in the male genitalia and larval head. Conversely, the tiny body and especially peculiar prothoracic structures indicate that *Foveapeltis*, although rather primitive in other morphological structures, was highly adapted to some specialized ecological niche and mode of life.

As we are not able to confidently determine the position of *Foveapeltis*, here we decide to leave it as Cleroidea *incertae sedis*. The most eye‐catching character of *Foveapeltis* is the presence of a large empty cavity on each hypomeron, which is unique among the whole Cleroidea. A somewhat similar hypomeral cavity is known in the monospecific family Acanthocnemidae (Matsumoto & Geiser, 2021). However, the cavity in Acanthocnemidae is covered by a sensory disc, serving as an infrared receptor (Kreiss et al., 2005; Schmitz et al., 2002; Zhou et al., 2016). The function of the empty cavity in *Foveapeltis* is presently unknown.

Extant adults of the trogossitid‐like groups are often mycophagous or predatory (Kolibáč & Leschen, 2010). Many of them are known to reside beneath tree bark or among the fruiting bodies of fungi. Decamerins are mostly floricolous and consume pollen grains. In our specimens, *Foveapeltis* adults are preserved along with a moth (Figure 9b), a platygastroid wasp (Figure 9c) or sand flies (Figure 9d), suggesting that they may actively fly or live in open spaces.

## AUTHOR CONTRIBUTIONS

**Yan‐Da Li:** Conceptualization (equal); data curation (equal); formal analysis (equal); investigation (lead); visualization (equal); writing – original draft (equal); writing – review and editing (lead). **Jiří Kolibáč:** Investigation (lead); writing – review and editing (lead). **Zhen‐Hua Liu:** Investigation (equal); writing – review and editing (equal). **Adam Ślipiński:** Investigation (equal); writing – review and editing (equal). **Shûhei Yamamoto:** Investigation (equal); writing – review and editing (equal). **Ya‐Li Yu:** Funding acquisition (equal); investigation (equal); writing – review and editing (equal). **Wei‐Ting Zhang:** Investigation (equal); writing – review and editing (equal). **Chen‐Yang Cai:** Conceptualization (equal); funding acquisition (equal); investigation (equal); supervision (equal); writing – review and editing (equal).

## CONFLICT OF INTEREST STATEMENT

The authors declare that they have no known competing financial interests or personal relationships that could have appeared to influence the work reported in this paper.

## Supporting information


File S1.

File S2.

File S3.

## Data Availability

The data matrix and R scripts for the phylogenetic analyses are available in the Supplementary material. The original confocal and micro‐CT data are available in the Zenodo repository (https://doi.org/10.5281/zenodo.11227307).
